# Effectiveness of Oral Iron Therapy in Anemic Inpatient Pregnant Women: A Single Center Retrospective Cohort Study

**DOI:** 10.7759/cureus.56879

**Published:** 2024-03-25

**Authors:** Claire Sutter, Robert E Freundlich, Britany L Raymond, Sarah Osmundson, Colleen Morton, David R McIlroy, Matthew Shotwell, Xiaoke Feng, Jeanette R Bauchat

**Affiliations:** 1 Anesthesiology, Vanderbilt University Medical Center, Nashville, USA; 2 Maternal Fetal Medicine, Vanderbilt University Medical Center, Nashville, USA; 3 Hematology, Vanderbilt University Medical Center, Nashville, USA; 4 Biostatistics, Vanderbilt University Medical Center, Nashville, USA

**Keywords:** pregnant, obstetric, iron deficiency, hospitalized, hemoglobin, anemia

## Abstract

Background and aim

Oral iron therapy is effective in treating iron deficiency anemia in outpatient pregnant women but has not been studied in inpatient pregnant women. We aimed to evaluate the effect of oral iron therapy versus no therapy during hospitalization on maternal and neonatal outcomes in women with anemia who are hospitalized for pregnancy-related morbidities (i.e., preterm premature rupture of membranes, preterm labor, pre-eclampsia, abnormal placentation, or fetal monitoring).

Methods

A retrospective, single-center study was conducted in hospitalized pregnant women (2018 to 2020) with inpatient stays of more than three days. The primary outcome was a change in hemoglobin level from admission to delivery in women treated with oral iron compared with those left untreated. Secondary outcomes included the total amount of iron administered before delivery, the time interval from admission to delivery, and neonatal effects.

Results

Two hundred sixty-three women were admitted, 79 women had anemia, and 29 (36.7%) received at least one dose of oral iron. Baseline patient characteristics were similar between groups. The median (interquartile range) dose of iron in the oral iron group was 1185.0 (477.0, 1874.0) mg. Neither absolute hemoglobin before delivery (control group: 10.0±1.2 g/dL; iron group: 10.1±1.1 g/dL; p=0.774) nor change in hemoglobin from admission to delivery (control group: -0.1±1.1 g/dL vs. iron group: 0.4±1.1 g/dL; p=0.232) differed between groups. Women in the control group had shorter length of stay (LOS) median (IQR) than women in the iron group (control group: 7.1 (5.0, 13.7) days; iron group: 11.4 (7.4, 25.9) days; p=0.03). There were no differences in maternal mode of delivery, though each group had high rates of cesarean delivery (control group: 53.7%; iron group: 72.4%; p=0.181). There were no differences in estimated blood loss at delivery (control group: 559±401; iron group: 662.1±337.4;p=0.264) in either group. Neonatal birthweight (control group: 1.9±0.7 kg; iron group: 1.9±0.7 kg; p=0.901), birth hemoglobin (control group: 16.3±2.2 g/dL; iron group: 16±2.2 g/dL; p=0.569), neonatal intensive care unit (NICU) admission (control group: 93.3%; iron group: 84.8%;p=0.272 ), or neonatal death (control group: 8.9%; iron group: 3%; p=0.394) were not different between groups.

Conclusions

Oral iron administered to anemic inpatient pregnant women was not associated with higher hemoglobin concentrations before delivery. Lack of standardized iron regimens and short hospital stays may contribute to the inefficacy of oral iron for this inpatient pregnant population. The small sample size and retrospective nature of this study are limiting factors in drawing conclusive evidence from this study.

## Introduction

Iron deficiency anemia (IDA) is the most common cause of anemia during pregnancy, affecting up to 12-30% of women in developed nations [1,2]. Despite presumed access to adequate nutrition in developed nations, over 40% of women have low iron reserves (defined as serum ferritin <30 ng/L).

Symptoms of peripartum IDA include fatigue and cardiovascular symptoms, while associated outcomes include increased risk of pre-eclampsia, preterm delivery, cesarean delivery, blood transfusion, prolonged hospitalization, and mortality [3-6]. Neonates similarly have adverse outcomes, including lower hemoglobin (Hb), iron stores, birthweight, APGAR (appearance, pulse, grimace, activity, and respiration) scores, and increased neonatal intensive care unit (NICU) admission, sepsis, and mortality [7,8].

Despite the myriad of potential adverse maternal and neonatal outcomes, the standard treatment for IDA in pregnancy continues to be oral (PO) iron because of cost, availability, and reported efficacy, but adherence to therapy is low due to side effects [9-11]. There remains a lack of consensus on diagnostic criteria in pregnancy, resulting in a lack of standardized dosing, frequency, and timing of iron therapy [12-14]. Furthermore, PO iron requires weeks to achieve maximal effects on Hb levels and months to replenish iron stores [15,16]. Given the high rates of IDA and the time required for treatment, hospitalized pregnant women may not benefit from oral iron therapy.

The primary objective of this study was to evaluate the change in Hb level between admission and delivery for inpatient pregnant women with anemia receiving PO iron compared with women left untreated. Secondary objectives included characterizing time from admission to delivery, total iron administered before delivery, and neonatal outcomes. We hypothesized that hospitalized pregnant women who received oral iron therapy would have higher Hb levels within 24 hours of delivery compared to those who did not receive PO iron.

Some initial findings of this study were previously presented as a five-minute oral presentation titled "Effect of Oral Iron Therapy on Hemoglobin Levels Prior to Delivery in Pregnant Inpatients with Anemia" at the Society for Obstetric Anesthesia and Perinatology 53rd annual meeting (virtual event), May 13-16, 2021.

## Materials and methods

Study design and study criteria 

The institutional review board at our institution approved this retrospective cohort study of anemic inpatient pregnant women admitted from March 1, 2018, to August 1, 2020 (IRB Number 200078). As it was a retrospective study design, there was no effect on clinical decision-making or patients' medical care. Women were eligible if they met the criteria for anemia in pregnancy (defined as Hb<11.0 g/dL at ≥28 weeks gestation or Hb<10.5 g/dL at <28 weeks gestation), were anticipated to be admitted for ≥3 days and delivered at our center during this hospital stay. Exclusion criteria included sickle cell disease and thalassemia, as their associated interventions may significantly alter hemoglobin levels. Women who received blood transfusion or intravenous (IV) iron administration during their hospitalization were also excluded for this reason. Only women who received oral iron were included since this is considered first-line therapy for iron deficiency anemia in the pregnant patient population [17]. Although serum ferritin <30 ng/L is used to diagnose IDA, ferritin tests and other iron studies were not available on all anemic patients. Therefore, acknowledging that iron deficiency is the most common cause of anemia during pregnancy, we included all women who met the diagnosis of anemia by hemoglobin levels alone. The difference in Hb level was defined as the change between admission Hb level (first Hb value within 24 hours of admission) and pre-delivery Hb level (lowest Hb value within 24 hours before delivery). Women receiving ≥1 dose of PO iron-specific therapy during hospitalization were categorized in the PO iron treatment group to capture all women who received iron therapy, though we would expect most women to receive >1 dose if they received the typical iron regimen for their entire hospitalization. The typical iron regimen prescribed at this institution is oral iron 325 mg twice a day with increasing or decreasing amounts per patient tolerance. Women receiving prenatal vitamins with iron were categorized as controls, so we calculated total elemental iron estimated from vitamin sources. We attempted to reduce selection and reporting bias by extracting data from impartial electronic health record (EHR) software using International Classification of Disease (ICD) 9 and 10 codes, and investigators manually validated key or missing variables.

Study procedure and assessments

Via EHR software, we identified 263 patients admitted to antepartum services for ≥3 days between March 1, 2018, and August 1, 2020. We randomly selected 10% of patients to verify data validity via manual chart review for variables pulled electronically.

Baseline demographics included age, height, weight, body mass index (BMI), American Society of Anesthesiologists Physical Status (ASA PS), and insurance status. Patients self-reported race and Hispanic ethnicity. Two investigators manually reviewed co-morbidities owing to the ambiguity of many ICD codes and the difficulty in capturing diagnostic lists through provider notes. Investigators manually reviewed each patient's admission diagnosis, and pregnancy-complicating conditions were documented.

Length of stay (LOS) and pregnancy-related data, including gestational age on admission and at delivery, maternal gravidity and parity, multiple gestation, use of IV magnesium, mode of delivery, estimated blood loss (EBL) at delivery, and medications used to treat atony or postpartum hemorrhage, were extracted from the EHR.

Lab values and iron administration data were extracted from the EHR and validated manually by investigators. Lab values of interest included Hb and iron studies. Investigators reviewed each patient's medication administration record for prenatal vitamin and iron administration data; the total amount of elemental iron and the total number of tablets administered during admission were collected. Neonatal data collected included Hb level at birth, APGAR scores at one and five minutes, birthweight, NICU admission, and mortality.

Sample size calculation

For a sample of 30 participants per group, there is about 95% power to detect a one g/dL difference between groups in the change in Hb value from baseline to delivery (standard deviation of 1.1 g/dL). This difference was deemed clinically significant since it could determine the need for an intervention such as a transfusion during the peripartum period.

Statistical analysis

Variables were summarized using mean ± standard deviation (SD) or median (IQR) for continuous variables and percentage for proportions. The Pearson chi-square test, Fisher exact test (when any cross-tabulated count was less than five), t-test, and Wilcoxon rank sum test were performed to make unadjusted comparisons across treatment and control groups. Within-group changes in Hb concentration from baseline to delivery were tested using a one-sample z-test. A propensity score weighting method was used to account for potential confounding of the association between PO iron therapy and outcomes. The propensity score for each patient was estimated using multivariable logistic regression, adjusting for BMI, age, length of hospital stay, insurance type, multiple gestation pregnancy, and gestational age at delivery. We then used the propensity score matching weights to weigh the contribution of each patient in the subsequent analysis [18]. Balance checking was done using the standardized mean difference between groups; successful weighting was demonstrated if the imbalance was improved in the patient factors and the standardized mean difference was less than 0.2. The adjusted effects of PO iron therapy on primary and secondary outcomes were examined using a propensity score weighted linear regression model for continuous outcomes and a propensity score weighted logistic regression model for binary outcomes. The PO iron therapy effect was summarized using an estimate (odds ratio or difference in mean) and a 95% confidence interval. A series of sensitivity analyses were performed; the analysis for primary outcome was repeated on patients who received a total of less than or equal to 500 mg of iron and on patients who received a total of greater than 500 mg of iron. We also adjusted for pre-eclampsia and pre-term premature rupture of membranes (PPROM), which could confound or mediate the effect of PO iron therapy on change in Hb level between admission and delivery. Participants with missing primary or secondary outcomes were omitted from the statistical analysis of those outcomes.

All analyses were performed using the R programming language 3.3.0 (R Foundation for Statistical Computing, Vienna, Austria). A p-value of <0.05 was considered a statistically significant difference.

## Results

Patient selection and demographics

**Figure 1:**
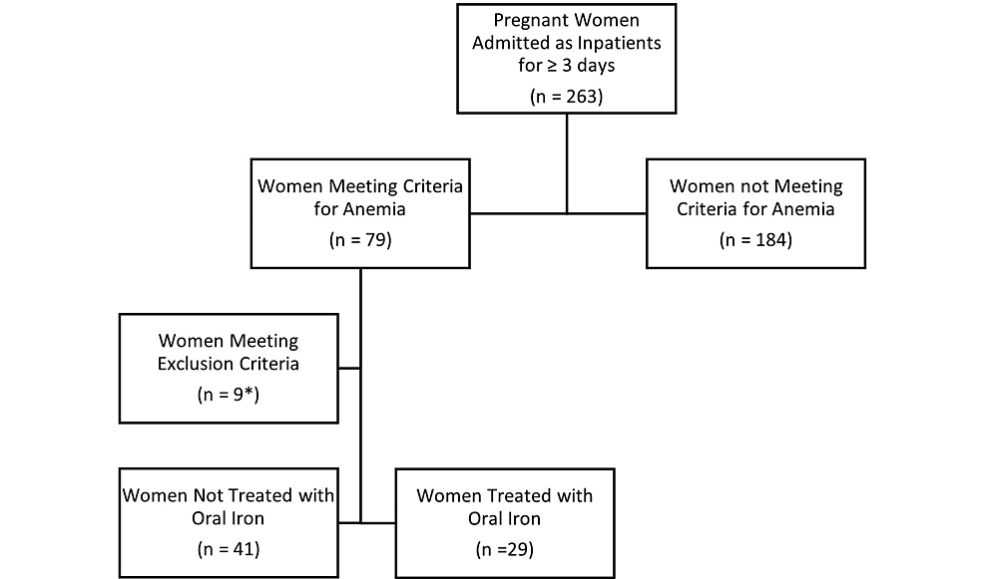
CONSORT flow diagram * five women were excluded from blood transfusion during hospitalization, and four women were excluded from intravenous iron administration during hospitalization. CONSORT - Consolidated Standards of Reporting Trials

Of 263 women admitted between 2017 and 2020, 79 met the criteria for diagnosis of anemia (30%). Nine were excluded owing to blood transfusion (n=5) or IV iron administration (n=4). Of the 70 women qualifying for the study, 29 received PO iron (41%). Although the control group (n=41, 58.6%) received no specific treatment for anemia, 65.9% (n=27) were administered prenatal vitamins. Missing data included admission Hb values (n=4) and delivery Hb values (n=24), as specified within the 24-hour time frame. For fifty-one women, no additional iron studies beyond a complete blood count (CBC) were recorded (Figure 1).

Women in both groups were similar in their co-morbidities and pregnancy characteristics (Table 1). The majority of the women were White (n=48), followed by Black (n=17). Only six women identified themselves as being of Hispanic ethnicity, and all were within the control arm (control group: 14.6%; iron group: 0.0%; p=0.038). A minority of women had private insurance (n=26, 37.1%). The most common admission diagnoses in each group were pre-eclampsia and PPROM. The most common co-morbidities included mood disorder, chronic hypertension, pulmonary disease, substance abuse, and use of tobacco products. Patients in the treatment group were more likely to have iron studies to characterize their anemia (control group: 14.6%; iron group: 44.8%; p=0.012). 

**Table 1:** Patient characteristics * Wilcoxon rank sum test; † Pearson chi-square test or Fisher exact test P-values indicate statistical significance at values <0.05. Race and Hispanic ethnicity, self-reported by patients upon admission and documented in the EHR, were verified manually by an investigator. Patients had the option to identify as American Indian/Alaska Native, Asian Indian, Black, unknown, unspecified Asian, or White. This variable was collected to ensure that neither group was vastly skewed towards one race or ethnicity since both factors are associated with differences in maternal morbidity and mortality. ASA - American Society of Anesthesiologists; IV - intravenous; EHR - electronic health record

Characteristic	No iron therapy (n=41)	Oral iron therapy (n=29)	p-value
Age (years)	28.9±5.5	30.0±6.8	0.440*
Race			0.181†
American Indian/Alaska Native	4.9% (n=2)	0.0% (n=0)	
Asian Indian	2.4% (n=1)	0.0% (n=0)	
Black	17.1% (n=7)	34.5% (n=10)	
Unknown	0.0% (n=0)	3.4% (n=1)	
Unspecified Asian	2.4% (n=1)	0.0% (n=0)	
White	73.2% (n=30)	62.1% (n=18)	
Ethnically Hispanic	14.6% (n=6)	0% (n=0)	0.038†
Height (inches)	63.2±3.4	64.2±3.5	0.299*
Weight (kg)	80.9±21.4	87.5±28.2	0.263*
BMI (kg/m^2^)	31.2±7.2	32.8±9.9	0.177*
ASA Physical Status Classification			0.482†
I	0.0% (n=0)	0.0% (n=0)	
II	39% (n=16)	27.6% (n=8)	
III	58.5% (n=24)	65.5% (n=19)	
IV	2.4% (n=1)	6.9% (n=2)	
Private insurance	36.6% (n=15)	37.9% (n=11)	1.00†
Gravida (number of times pregnant)	3.7±2.7	2.9±2.2	0.210*
Parity (number of births)	1.9±1.9	1.3±1.6	0.204*
Multiple gestation pregnancy	12.2% (n=5)	13.8% (n=4)	1.000†
Gestational age (weeks) at admission	30.2±3.6	29.5±4.0	0.455*
Gestational age (weeks) at delivery	31.8±3.4	32.1±3.7	0.736*
Length of hospital stay, admission-delivery (days)	11.0±10.4	18.1±15.4	0.024*
Admission diagnosis			
Pre-eclampsia	22.0% (n=9)	34.5% (n=10)	0.374†
Preterm premature rupture of membranes	39.0% (n=16)	20.7% (n=6)	0.172†
Preterm labor	14.6% (n=6)	10.3% (n=3)	0.726†
Placenta previa	4.9% (n=2)	10.3% (n=3)	0.642†
Vasa previa	0.0% (n=0)	6.9% (n=2)	0.168†
Fetal reasons	4.9% (n=2)	10.3% (n=3)	0.642†
Maternal co-morbidities	14.6% (n=6)	10.3% (n=3)	0.726†
Administration of IV magnesium during admission	43.9% (n=18)	58.6% (n=17)	0.332†
Additional anemia labs were drawn during admission	14.6% (n=6)	44.8% (n=13)	0.012†
Average serum ferritin (ng/mL)	17.7±11.1	19.1±16.5	0.857*
Average serum total iron binding capacity ug/dL)	377.2±49.5	445.8±55.3	0.058*
Average serum iron level (ug/dL)	65.0±35.6	93.3±91.4	0.543*

Primary outcome

There was no change between admission and delivery Hb values within the control group (p=0.677), iron group (p=0.081), or between these groups (Table 2). In the unadjusted analysis, the incidence of anemia at the time of delivery was not different between groups. However, admission Hb levels were lower in the iron group.

**Table 2:** Outcomes data * t-test; † Pearson chi-square test or Fisher exact test P-values indicate statistical significance at values <0.05. NICU - neonatal intensive care unit; APGAR - appearance, pulse, grimace, activity, and respiration

Outcome	No iron therapy (n=41)	Oral iron therapy (n=29)	p-value
Admission hemoglobin (g/dL)	10.2±0.6	9.7±0.9	0.004*
Delivery hemoglobin (g/dL)	10.0±1.2	10.1±1.1	0.774*
Change in hemoglobin (g/dL)	-0.1±1.1	0.4±1.1	0.232*
P-values of change in Hb within each arm	0.677	0.081	
Anemic at the time of delivery	82.6% (n=19)	60.9% (n=14)	0.189†
Delivery by cesarean section	53.7% (n=22)	72.4% (n=21)	0.181†
Estimated blood loss during delivery (mL)	559.1±401.2	662.1±337.4	0.264*
Medications and blood products required during delivery			
Misoprostol	29.3% (n=12)	44.8% (n=13)	0.278†
Carboprost	12.2% (n=5)	13.8% (n=4)	1.000†
Methylergonovine	9.8% (n=4)	27.6% (n=8)	0.062†
Red blood cell transfusion	4.9% (n=2)	6.9% (n=2)	1.000†
Fresh frozen plasma transfusion	0.0% (n=0)	3.4% (n=1)	0.414†
Cryoprecipitate administration	0.0% (n=0)	0.0% (n=0)	
Fibrinogen concentrate administration	0.0% (n=0)	3.4% (n=1)	0.414†
Neonatal outcomes	(n=46)	(n=33)	
Birthweight (kg)	1.9 ± 0.7	1.9±0.7	0.901*
Birth hemoglobin (mg/dL)	16.3±2.2	16.0±2.2	0.569*
1st APGAR score	6.5±2.2	6.5±2.4	0.997†
2nd APGAR score	7.7±2.2	7.5±2.1	0.797†
NICU admission	93.3% (n=42)	84.8% (n=28)	0.272†
Neonatal death	8.7% (n=4)	3.0% (n=1)	0.394†

**Figure 2:**
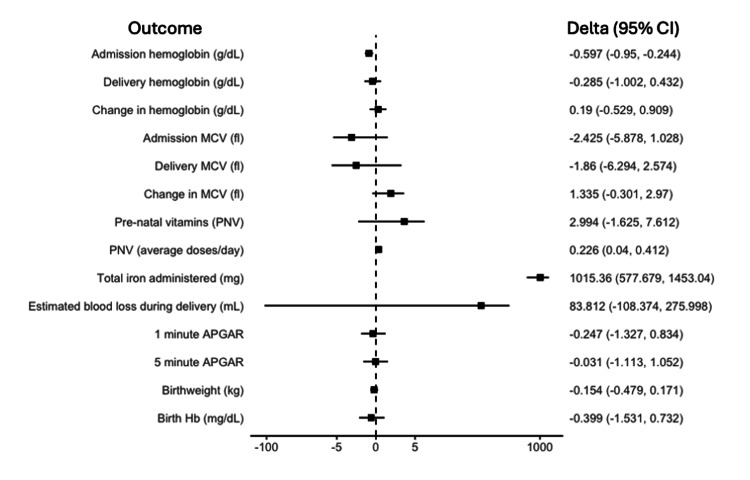
Mean differences in hematological, delivery, and neonatal outcomes CI - confidence interval; MCV - mean corpuscular volume; APGAR - appearance, pulse, grimace, activity, and respiration (score from 0 to 10)

**Figure 3:**
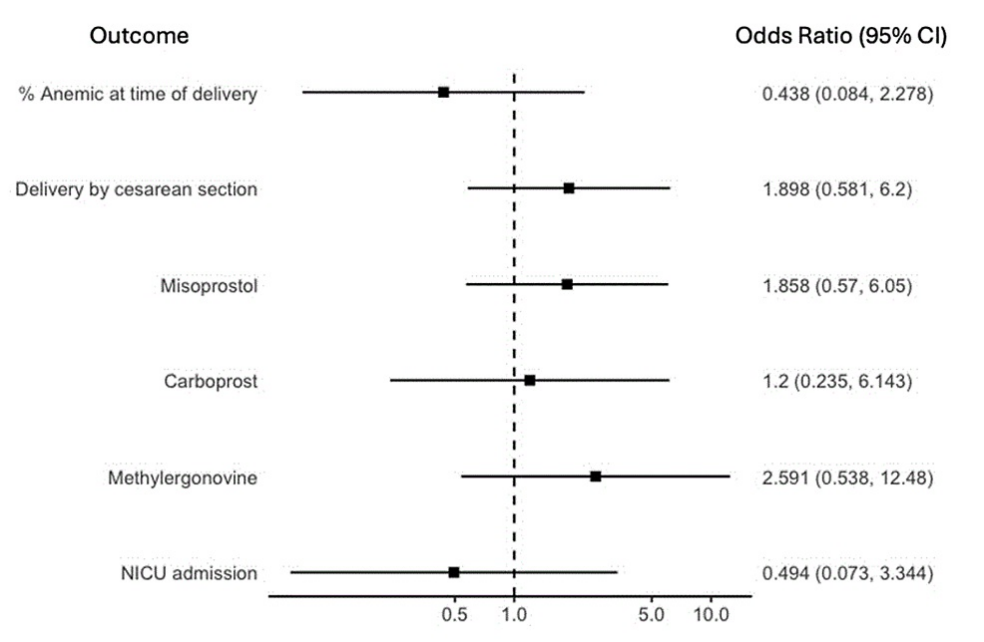
Odds ratios of hematological, delivery, and neonatal outcomes CI - confidence interval; NICU - neonatal intensive care unit

After propensity score weighted matching, the standardized mean differences for adjusted covariates were all less than 0.2, indicating a balance between treatment and control groups. In the adjusted analysis, PO iron therapy was associated with lower average admission Hb level (difference in means: -0.597, CI: -0.950, -0.244; p=0.001). There was no effect of PO iron on average change in Hb value between admission and delivery (difference in means: 0.190, CI: -0.529, 0.909; p=0.597), on average change in mean corpuscular volume MCV between admission and delivery (difference in means: 1.335, CI: -0.301, 2.970; p=0.107), or on incidence of anemia at delivery (odds ratio: 0.438, CI: 0.084, 2.278; p=0.326; see Figures 2-3).

Secondary outcomes

The incidence of prenatal vitamin therapy was higher for women in the iron group compared to controls (control group: 65.9%; iron group: 93.1%; p=0.011). The median total amount of iron administered during admission was higher in the iron group (control group: 447 mg (0, 232); iron group: 1874 mg (477, 1874); p<0.001). The median (IQR) number of iron tablets administered throughout the treatment groups' stay was 14 (5, 21), with a frequency of one (0.7, 1.5) tablets per day. The average amount of elemental iron per day was calculated using total iron dose and length of stay (control group: 15.5 (0, 25.9); iron group: 85.7 (58.4, 123.4); p<0.001). In the adjusted analysis, PO iron therapy was associated with increased iron administration (difference in means: 1015.36, CI: 577.679, 1453.040; p<0.001) and was associated with an increase in the average number of prenatal vitamins administered per day (difference in means: 0.226, CI: 0.040, 0.412; p=0.018). There was no effect of PO iron therapy on the average total of prenatal vitamins administered (difference in means: 2.994, CI: -1.625, 7.612, p=0.200).

Secondary outcomes related to maternal delivery and neonatal data demonstrated no differences between groups (Figures 2-3). In both groups, the majority of women underwent cesarean delivery with similar estimated blood loss. There were no differences in the administration of uterotonics or blood products. Women in the control group had shorter LOS median (IQR) than women in the iron group (control group: 7.1 (5.0, 13.7) days; iron group: 11.4 (7.4, 25.9) days; p=0.03). Neonatal outcomes demonstrated no differences in birthweight, birth Hb level, one-minute or five-minute APGARs, NICU admission, or mortality (Table 2). The adjusted analysis showed no effect of PO iron on secondary outcomes related to maternal delivery and neonatal data (Figures 2-3).

Sensitivity analysis

**Figure 4:**
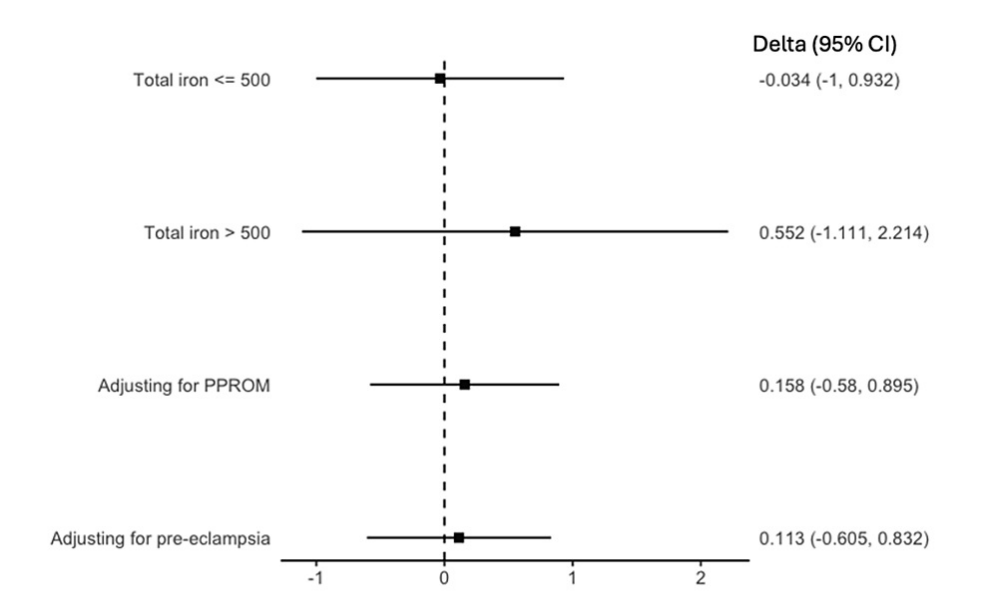
Sensitivity analysis CI - confidence interval; PPROM - preterm premature rupture of membranes

In the sensitivity analysis, there was no effect of PO iron on the average change in Hb value between admission and delivery for patients receiving less than or equal to 500 mg of iron (difference in means: -0.034, CI: -1.000, 0.931; p=0.943) or for patients receiving greater than 500 mg total of iron (difference in means: 0.552, CI: -1.111, 2.214; p=0.943). There was no effect of PO iron on the average change in Hb value between admission and delivery after adjusting for pre-eclampsia (difference in means: 0.158, CI: -0.580, 0.895; p=0.668) or PPROM (difference in means: 0.113, CI: -0.605, 0.832; p=0.752; see Figure 4).

## Discussion

Principal findings

This single-center study demonstrates that anemia is common, with an incidence of approximately 30% of women admitted for pregnancy-related complications. However, anemia is rarely fully characterized and is likely undertreated in a hospital setting without standardized anemia treatment guidelines. Hospitalized women at our institution only remained pregnant for an additional one to three weeks, so when treated for anemia and presumed IDA with PO iron, they had no change in Hb level from admission to pre-delivery and no detectable outcome improvements compared with those untreated. Women receiving PO iron had longer predelivery hospital stays and, therefore, gestation, but it is unclear whether this was due to the oral iron therapy.

According to the American College of Obstetricians and Gynecologists, the first line therapy for the treatment of pregnant women with IDA is oral iron therapy unless they have not tolerated oral iron therapy, have not responded to it, or have severe anemia late in pregnancy [17]. Women in the iron group received approximately 10 times more iron during their hospital course than the control group but did not have a higher Hb level by delivery. Women receiving iron-specific therapy likely did not see benefit from therapy similar to outpatient studies because of the short duration of hospital stay, which is unpredictable at the time of hospital admission. Multiple studies comparing the effects of PO iron versus placebo in the outpatient setting have found a change in Hb level of 1-2 g/dL over approximately 20 weeks, which is unrealistic in this patient population [19-25] (see appendix). In several studies, there was little to no benefit in giving more than 30-65 mg of elemental iron per day to pregnant women, and it may be as beneficial to give every other day dosing [20-21,24,26-27]. The iron group in our study achieved close to 85 mg/day of elemental iron, yet no rise in Hb level was detected, likely related to the unanticipated short duration of therapy.

Those with anemia demonstrated risk factors of low socioeconomic status, substance use, and limited or no prenatal care, likely predisposing them to poor access to nutrition and PO iron supplementation. Rapid replacement of iron stores in these high-risk hospitalized patients while within the healthcare system may require IV iron therapy, as our study demonstrated there is insufficient time for PO iron to be efficacious. Additionally, we found a very high rate of cesarean delivery (61.4%) among these patients; thus, treatment of IDA is more important because of higher expected EBL during cesarean versus vaginal delivery.

Clinical and research implications

This study has several implications, including the need for standardized identification and treatment of anemia among pregnant inpatients, given the inconsistency and variability in anemia work-up and treatment even within one institution. Standardization of practice for hemorrhage and pre-eclampsia is well-established and leads to a marked reduction in maternal morbidity and mortality [28]. A standard proactive approach to anemia could be similarly beneficial, as encouraged by the World Health Organization through patient blood management programs. Even though our overall numbers are low, no women of Hispanic ethnicity were given PO iron in our study, and disparities in obstetric outcomes in Hispanic and other minority populations are well-known, and standardization may increase care equity [29]. Future studies in this inpatient population should focus on the optimization of outcomes, including rapid escalation to IV iron, since studies demonstrate that IV iron may be more efficacious than PO iron in increasing Hb levels in obstetric settings [30,31].

Strengths and limitations

Strengths of this study include a focus on well-matched cohort participants who are not frequently studied but who have prolonged inpatient stays at many tertiary care facilities. This study was also able to pull both maternal and short-term neonatal outcome data. Limitations include the retrospective nature of the study, the small sample size, the limited ability to identify pre-admission iron regimens, the infrequent ordering of iron studies, non-standardized oral iron regimens, and no long-term outcomes. Lastly, patient-centered subjective data such as fatigue could not be collected.

## Conclusions

In conclusion, many studies demonstrate the efficacy of outpatient PO iron in correcting IDA of pregnancy. In hospitalized pregnant women with anemia, our study demonstrated that fewer than three weeks are available for the treatment of anemia before delivery. Therefore, PO iron treatment of anemia in hospitalized pregnant women is likely ineffective, and investigation into alternative strategies is warranted. These conclusions may not be generalizable and are limited by the small sample size and retrospective, single-center nature of this study. 

## Appendices

**Table 3:** Iron regimens from previous studies Data modified from [19].

Iron supplementation regimen (regimen started, Hgb measured)	Effect on hemoglobin level
105 mg iron daily (16 weeks, 36 weeks)	0.4 g/dL
30 mg iron daily (20 weeks, 37 weeks)	1.0 g/dL
100 mg iron twice a day (11-14 weeks, 35 weeks)	1.0 g/dL
65 mg iron daily (16 weeks, 35-40 weeks)	1.0 g/dL
200 mg iron daily (10-12 weeks, 37-40 weeks)	1.3 g/dL
65 mg iron with folate daily (12 weeks, 36 weeks)	1.6 g/dL
100 mg sustained release iron twice daily (16 weeks, 36 weeks)	1.7 g/dL
